# The effect of pediatric acute potassium chloride correction infusions on the serum potassium concentration

**DOI:** 10.62675/2965-2774.20240113-en

**Published:** 2024-10-11

**Authors:** Jade Miller, Alex Dewar, Andrew Wignell, Patrick Davies

**Affiliations:** 1 University of Nottingham School of Medicine Nottingham United Kingdom School of Medicine, University of Nottingham - Nottingham, United Kingdom.; 2 Nottingham Children's Hospital Paediatric Critical Care Unit Nottingham United Kingdom Paediatric Critical Care Unit, Nottingham Children's Hospital - Nottingham, United Kingdom.; 3 Nottingham Children's Hospital Pharmacy Department Nottingham United Kingdom Pharmacy Department, Nottingham Children's Hospital - Nottingham, United Kingdom.

## INTRODUCTION

In pediatric intensive care unit (ICU), serum potassium concentrations within the normal range promotes more stable physiology.^(1-4)^ A common treatment for hypokalemia in pediatric ICU is potassium ‘correction’, an intravenous infusion of 0.4mmol/kg (maximum 20mmol) potassium chloride 0.5mmol/mL over 1 hour. Patients with a serum potassium concentration < 3mmol/L are typically treated unless they have specific conditions, e.g., cardiac arrhythmia, requiring higher correction thresholds. Concentrated potassium correction protocols vary among pediatric ICUs in the United Kingdom, with doses usually ranging from 0.4 - 0.5mmol/kg/hour.

The effects of such corrections have been studied only in adults.^(5-9)^ We aimed to study the effectiveness of potassium chloride correction infusions at correcting hypokalemia in children and the risk of "overshooting" and causing hyperkalemia.

## METHODS

We examined all intravenous potassium chloride correction infusions over 4.5 years in a tertiary pediatric ICU. All patients underwent continuous electrocardiogram monitoring. We recorded the serum concentrations of potassium, glucose, sodium, magnesium, and bicarbonate and the pH preinfusion and postinfusion. Outliers from sampling errors (e.g., hemolysis, confirmed by a second, markedly lower potassium concentration shortly after infusion) were excluded. This was assessed by an independent clinician.

Patients on renal replacement therapy or with syndromes involving excessive potassium loss, e.g., Bartter's syndrome, were excluded. The effects of the preinfusion parameters on the increase in the potassium concentration was determined via linear regression. Descriptive statistics and 95% confidence intervals (95%CI) were calculated.

Ethical approval was not needed, as this was classified as a service evaluation by the NHS Health Research Authority.

## RESULTS

**Figure 1:**
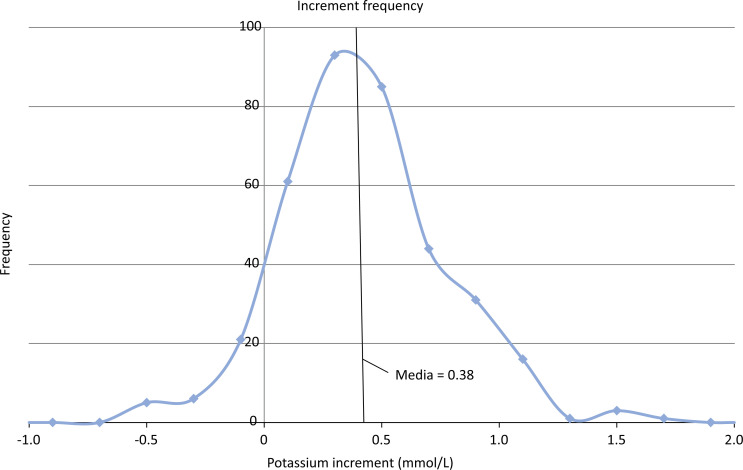
Frequency of serum potassium concentration increases (mmol/L) after a one-hour intravenous infusion of 0.4mmol/kg potassium chloride.

One hundred thirty-five patients received 369 correction infusions (220 males, 149 females). The mean ± standard deviation (SD) preinfusion potassium concentration was 2.71 (± 0.33) mmol/L. The mean ± SD age and weight were 4.79 years (± 4.93) and 16.9 kg (± 12.7), respectively. The main diagnostic categories were sepsis (22,7%), respiratory disease (22.5%), oncological disease (10.3%), acute renal disease (8.8%), and gastrointestinal disease (7.7%). The mean ± SD increase in the potassium concentration was 0.38 (± 0.36) mmol/L (95%CI 0.34 to 0.41), indicating a concentration/dose ratio of 0.95. Fourteen (3.8%) patients had an increase in the potassium concentration > 1mmol/L: 6 (1.6%) had an increase > 1.2mmol/L, and 3 (0.81%) had an increase > 1.5mmol/L (Figure 1). The maximum postinfusion serum potassium concentration was 4.60mmol/L. In 32 (8.7%) patients, the serum potassium decreased concentration following infusion, with a maximum decrease of −0.60mmol/L.

Linear regression and R^2^ values revealed no correlation among preinfusion potassium, glucose, sodium, bicarbonate, or magnesium concentration, pH, age or weight, and increase in the potassium concentration (all R^2^ values < 0.0035). The time between infusion completion and sampling was not associated with the level of the potassium concentration for a minimum of three hours postinfusion.

The mean ± SD increase in the potassium concentration was 0.36mmol/L (± 0.43) for females and 0.39mmol/L (± 0.44) for males, indicating no significant difference. There was no association between the increase in the potassium concentration and diagnostic category.

## DISCUSSION

Our results suggest that, on average, administering 0.4mmol/kg intravenous potassium chloride over one hour increases the serum potassium concentration by nearly 0.4mmol/L, which is sustained for at least 3 hours.

The magnitude of the potassium increase was not associated with any of the measured factors, reinforcing dose predictability. Even among the outliers, there were no cases of hyperkalemia (> 5.3mmol/L) postinfusion. The decrease in the serum potassium concentration following infusion may be attributed to significant ongoing losses.

Comparisons with previous adult studies^(5-9)^ are challenging because of methodological heterogeneity and physiological differences between adults and children.

Concomitant administration of potassium (e.g., in maintenance fluids) were not included in the analysis; however, this reflects standard practice in such patients. We were unable to analyze the effects beyond 3 hours postinfusion, as other interventions and changing clinical conditions would have affected the results. Data on renal function were excluded because of the inability to accurately describe the eGFR in pediatric critical care patients; however, patients with both acute and chronic renal failure showed similar increases to those in patients without renal failure.

Our study therefore highlights the predictability and safety of administering concentrated potassium chloride infusions.
