# Supplementary material for: The effect of pediatric acute potassium chloride correction infusions on the serum potassium concentration
Source: Crit Care Sci. 2024 Oct 11;36:e20240113en. doi: 10.62675/2965-2774.20240113-en (PMC11554296; doi:10.62675/2965-2774.20240113-en)
Supplement: Translation

# Translation: Efeito das infusões de correção aguda de cloreto de potássio em pediatria na concentração sérica de potássio

**DOI:** 10.62675/2965-2774.20240113-pt

**Authors:** Jade Miller, Alex Dewar, Andrew Wignell, Patrick Davies

**Affiliations:** 1 Nottingham United Kingdom School of Medicine, University of Nottingham - Nottingham, United Kingdom.; 2 Nottingham United Kingdom Paediatric Critical Care Unit, Nottingham Children's Hospital - Nottingham, United Kingdom.; 3 Nottingham United Kingdom Pharmacy Department, Nottingham Children's Hospital - Nottingham, United Kingdom.

## INTRODUÇÃO

Na unidade de terapia intensiva (UTI) pediátrica, as concentrações séricas de potássio dentro da faixa normal promovem uma fisiologia mais estável.^(1-4)^ Um tratamento comum para hipocalemia na UTI pediátrica é a "correção" de potássio, uma infusão intravenosa de cloreto de potássio de 0,4mmol/kg (máximo de 20mmol) e 0,5mmol/mL durante 1 hora. Pacientes com concentração sérica de potássio < 3mmol/L são tipicamente tratados, a menos que tenham condições específicas, por exemplo, arritmia cardíaca, que exigem limites de correção mais alto. Os protocolos de correção de potássio concentrado variam entre as UTIs pediátricas no Reino Unido, com doses que geralmente variam de 0,4 a 0,5mmol/kg/hora.

Os efeitos de tais correções foram estudados apenas em adultos.^(5-9)^ Nosso objetivo foi estudar a eficácia das infusões de correção de cloreto de potássio para corrigir a hipocalemia em crianças e o risco de exceder os níveis de potássio e causar hipercalemia.

## MÉTODOS

Examinamos todas as infusões intravenosas de correção com cloreto de potássio al longo de 4,5 anos em uma UTI pediátrica terciária. Todos os pacientes foram submetidos a monitoramento contínuo por eletrocardiograma. Registramos as concentrações séricas de potássio, glicose, sódio, magnésio e bicarbonato e o pH antes e depois da infusão. Foram excluídos os casos atípicos de erros de amostragem (por exemplo: hemólise, confirmada por uma segunda concentração de potássio nitidamente mais baixa logo após a infusão). Isso foi avaliado por um clínico independente.

Pacientes em terapia de substituição renal ou com síndromes que envolvem perda excessiva de potássio, por exemplo, síndrome de Bartter, foram excluídos. Os efeitos dos parâmetros de pré-infusão sobre o aumento da concentração de potássio foram determinados por meio de regressão linear. Foram calculados as estatísticas descritivas e os intervalos de confiança de 95% (IC95%).

A aprovação ética não foi necessária, pois o estudo foi classificado como uma avaliação de serviço pela *NHS Health Research Authority*.

## RESULTADOS

Cento e trinta e cinco pacientes receberam 369 infusões de correção (220 do sexo masculino, 149 do sexo feminino). A concentração média ± desvio-padrão (DP) de potássio antes da infusão foi de 2,71 (± 0,33) mmol/L. A idade e o peso médios ± DP foram de 4,79 anos (± 4,93) e 16,9 kg (± 12,7), respectivamente. As principais categorias diagnósticas foram sepse (22,7%), doença respiratória (22,5%), doença oncológica (10,3%), doença renal aguda (8,8%) e doença gastrintestinal (7,7%). O aumento médio ± DP na concentração de potássio foi de 0,38 (± 0,36) mmol/L (IC95% 0,34 a 0,41), indicando relação concentração/dose de 0,95. Quatorze (3,8%) pacientes tiveram um aumento na concentração de potássio > 1mmol/L: 6 (1,6%) tiveram um aumento > 1,2mmol/L e 3 (0,81%) tiveram um aumento > 1,5mmol/L (Figura 1). A concentração máxima de potássio sérico pós-infusão foi de 4,60mmol/L. Em 32 (8,7%) pacientes, a concentração sérica de potássio diminuiu após a infusão, com diminuição máxima de −0,60mmol/L.

**Figura 1:** Frequência do aumento da concentração sérica de potássio (mmol/L) após infusão intravenosa de 1 hora de cloreto de potássio 0,4mmol/kg.

A regressão linear e os valores de R^2^ não revelaram correlação entre as concentrações de potássio, glicose, sódio, bicarbonato ou magnésio pré-infusão, pH, idade ou peso e o aumento na concentração de potássio (todos os valores de R^2^ < 0,0035). O tempo entre a conclusão da infusão e a coleta da amostra não foi associado ao nível da concentração de potássio por um mínimo de 3 horas após a infusão.

O aumento médio ± DP na concentração de potássio foi de 0,36mmol/L (± 0,43) para mulheres e 0,39mmol/L (± 0,44) para homens, indicando que não houve diferença significativa. Não houve associação entre o aumento na concentração de potássio e a categoria diagnóstica.

## DISCUSSÃO

Nossos resultados sugerem que, em média, a administração de 0,4mmol/kg de cloreto de potássio intravenoso ao longo de 1 hora aumente a concentração de potássio sérico em quase 0,4mmol/L, o que se mantém por pelo menos 3 horas.

A magnitude do aumento do potássio não esteve associada a nenhum dos fatores medidos, reforçando a previsibilidade da dose. Mesmo entre os valores discrepantes, não houve casos de hipercalemia (> 5,3mmol/L) após a infusão. A diminuição na concentração de potássio sérico após a infusão pode ser atribuída a perdas contínuas significativas.

As comparações com estudos anteriores em adultos^(5-9)^ são desafiadoras devido à heterogeneidade metodológica e a diferenças fisiológicas entre adultos e crianças.

Administração concomitante de potássio (por exemplo, em fluidos de manutenção) não foi incluída na análise; no entanto, isso reflete a prática-padrão nesses pacientes. Não pudemos analisar os efeitos além de 3 horas após a infusão, pois outras intervenções e mudanças nas condições clínicas poderiam ter afetado os resultados. Os dados sobre a função renal foram excluídos devido à impossibilidade de descrever com precisão a taxa de filtração glomerular estimada em pacientes pediátricos em cuidados críticos; no entanto, pacientes com insuficiência renal aguda e crônica mostraram aumentos semelhantes aos de pacientes sem insuficiência renal.

Nosso estudo, portanto, destaca a previsibilidade e a segurança da administração de infusões concentradas de cloreto de potássio.
